# Interstitial cystitis—an imbalance of risk and protective factors?

**DOI:** 10.3389/fpain.2024.1405488

**Published:** 2024-05-09

**Authors:** Jodi L. Westropp, Judith L. Stella, C. A. Tony Buffington

**Affiliations:** ^1^ Department of Medicine and Epidemiology, UC Davis School of Veterinary Medicine, Davis, CA, United States; ^2^ Department of Comparative Pathobiology, Purdue University College of Veterinary Medicine, W. Lafayette, IN, United States

**Keywords:** urinary bladder, chronic primary pain, chronic pelvic pain, bladder pain syndrome, biopsychosocial model, one health, cats, animal models

## Abstract

Interstitial cystitis (IC) presents as a chronic pain condition with variable combinations of symptoms depending on the species and individual patient. It is diagnosed by the presence of lower urinary tract signs and symptoms in combination with a variety of comorbid health problems, a history of life adversities, and the absence of other conditions that could cause the lower urinary tract signs. IC occurs naturally in humans and cats as a dimensional condition, with patients presenting with mild, moderate, and severe symptoms. Most patients appear to recover without specific treatment. A number of rodent models of IC have been used to study its causes and treatments. Unfortunately, current therapies generally fail to ameliorate IC symptoms long-term. The recent classification of IC as a chronic *primary* pain disorder calls for a rethinking of current clinical and research approaches to it. Beginning when a patient encounters a clinician, precipitating, perpetuating, and palliating risk factors can be addressed until a cause or reliably effective therapy is identified, and identifying predisposing and preventive factors can inform epidemiological studies and health promotion interventions. Predisposing, precipitating, and perpetuating risk factors, including environmental, psychological, and biological, increase the activity of the central threat response system (CTRS), which plays a clinically important role in IC symptoms. Studies in cats and rodent models have revealed that environmental enrichment (EE), in the absence of bladder-directed therapies, leads to amelioration of IC symptoms, implying a central role for the CTRS in symptom precipitation and perpetuation. Conceptually moving the source of IC pain to the brain as a motivational state rather than one resulting from peripheral nociceptive input offers both clinicians and researchers novel opportunities to improve care for patients with IC and for researchers to use more ecologically valid rodent models. It may even be that IC results from an excess of risk to protective factors, making this imbalance a targetable cause rather than a consequence of IC.

## Introduction

Interstitial Cystitis (IC) presents as a chronic painful condition with variable combinations of social, psychological, and biological problems depending on the species and individual patient. In addition to afflicting humans, IC occurs commonly in pet cats (*Felis catus*), is studied in rodent models, and has been reported sporadically in other mammals, including cheetahs, dogs, llamas, and pot-bellied pigs (1–3). This article will focus mainly on humans, cats, a naturally occurring model for IC in humans, and induced rodent models of IC because there is a paucity of information available about other species.

Chronic pain is defined by the International Association for the Study of Pain (IASP) as an unpleasant sensory and emotional experience associated with, or resembling that associated with, actual or potential tissue damage that persists or recurs for longer than three months^1^. The IASP has long recognized two classes of chronic pain: nociceptive pain, which results from tissue damage that doesn't heal, and neuropathic pain, which results from nerve injury. In 2019, the IASP classified IC as a chronic primary pain condition, which they define as one persisting for longer than three months, associated with significant emotional distress, functional disability, or a combination of the two, and not better accounted for by another condition (4). The IASP's 2019 revised chronic pain classification, although still controversial (5, 6), affords an opportunity to rethink current clinical and research approaches to IC.

### Patient presentation

Humans and cats with IC present similarly (Table 1).

**Table 1:** Comparison of results of studies in humans and cats with IC [adapted from (7) and updated].

Parameter		Human beings (3, 8)	Cats
Patient features			
1	Gender/Sex	2:1 Female: male (9)	Female: male varies (10, 11)
2	Genetic predisposition	Yes (12)	Yes (10, 11)
3	Early life adversities	Yes (13, 14)	Yes (15)
4	Recent life adversities	Yes (14)	Yes (16)
5	Bladder symptoms—frequency, urgency, pain	Yes (70)	Yes (1)
6	Nonbladder symptoms	Yes (1)	Yes (16, 18)
7	Waxing and waning clinical course	Yes (19)	Yes (1)
Comorbid disorders			
8	Gastrointestinal	Yes (20, 21)	Yes (16, 18)
9	Cardiovascular	Yes (20)	Yes (22)
10	Neurological	Yes (20, 21)	Yes (23, 24)
11	Psychological/behavioral	Yes (21)	Yes (18, 25)
Peripheral abnormalities			
Bladder			
12	Hunner lesions	Yes (5%–57%) (26)	Yes (rare) (27)
13	Increased urothelial permeability	Yes (28)	Yes (29)
14	Urothelial cell abnormalities	Yes (30)	Yes (29)
15	Increased mast cells	Yes (Hunner's ulcer) (31)	Yes (degranulated) (32)
16	Urine glycosaminoglycan excretion	Variable (33)	Yes (34)
17	Decreased glycosaminoglycan GP-51 expression	Yes (35)	Yes (36)
18	Vasodilatation and edema ± inflammatory infiltrate	Yes (37)	Yes (1, 32)
Sensory			
19	Increased bladder substance P immunoreactivity	Yes (38)	Yes (39)
20	Sensory neuron abnormalities	Yes (40)	Yes (41)
21	Dorsal root ganglia abnormalities	ND*	Yes (23)
22	Increased bladder substance P receptors	Yes (42)	Yes (39)
Central abnormalities			
23	Increased response to environmental stressors	Yes (43)	Yes (16)
24	Increased startle responsiveness	Yes (44)	Yes (45)
25	Increased locus coeruleus tyrosine hydroxylase immunoreactivity	ND	Yes (46)
Autonomic			
26	Increased plasma catecholamine concentrations	Yes (47)	Yes (48)
27	Increased urine norepinephrine excretion	Yes (47, 49)	ND
28	Increased bladder tyrosine hydroxylase immunoreactivity	Yes (50)	Yes (51)
29	Decreased alpha-2 adrenoceptor function	ND	Yes (52)
30	Increased bladder norepinephrine content	ND	Yes (53)
31	Decreased high frequency heart rate variability	Yes (54)	ND
32	Decreased parasympathetic activity	Yes (54)	ND
Endocrine			
33	Adrenocortical hormone abnormalities	Yes (14)	Yes (15)
34	Gonadal hormone abnormalities	Yes (55)	Yes (most neutered) (56)
Immune			
35	Immune cell and cytokine abnormalities	Yes (14, 57)	Yes (32)
36	Chronic inflammation	Yes (58)	Yes (59)
Response to treatment			
37	Amitriptyline	Very low certainty (60)	Uncontrolled trial (61)
38	Psychological/behavioral	Limited effectiveness (60)	Effective (62)

* ND, not determined to the authors’ knowledge.

Commonalities between IC in humans and cats include variable severity, a waxing & waning course, sensitivity to threatening environmental contexts, various peripheral and central abnormalities, and a variety of comorbidities. In humans, these comorbidities are called chronic overlapping pain conditions (COPC), which include chronic low back pain, chronic prostatitis, painful endometriosis, fibromyalgia, irritable bowel syndrome, migraine/tension-type headache, myalgic encephalomyelitis/chronic fatigue syndrome, temporomandibular disorder, and vulvodynia (17). In cats, comorbidities are called sickness behaviors (SB), which include variable combinations of gastrointestinal, cardiovascular, and dermatologic signs, elimination abnormalities, decreased food intake, and behavioral problems (16). Furthermore, bladder-targeted therapies (e.g., glycosaminoglycans, intravesicular resiniferatoxin, bladder hydrodistension, surgery, and special diets) have minimal long-term beneficial effects for most humans and cats.

IC in humans and cats also appears to be a dimensional rather than a categorical condition; patients may have minimal, mild, moderate, or severe symptoms that can vary over time rather than simply being present or absent. This has been studied most commonly in women (63–65). IC is also dimensional in cats, and more commonly severe in males because they can develop urethral obstruction (66).

After centuries of investigation, research and clinical articles still commonly state that the cause of IC remains unknown. Cause has a disputed history in philosophy and medicine however (67, 68), and recent experience with the global SARS-CoV-2 pandemic underscores the importance of pathogen, person, and place (environmental) factors in the outcome of diseases, even those with known causes. Moreover, ignorance of a cause need not prevent effective action. For example, citrus fruit was known to prevent scurvy for more than 500 years before the cause, ascorbic acid deficiency, was identified (69). This review aims to expand and extend the risk and protective factor approach (15, 16, 70) to IC to suggest opportunities to improve care and research while the search for a cause continues.

### Nosology

The classification and naming of IC, its nosology, has become complex and controversial in urology. Diseases may be classified by their presenting signs and symptoms, affected organ system(s), pathogenesis, and etiology (1). A significant challenge to accurate nosology exists because diseases may be named based on prominent signs and symptoms long before research identifies their pathogenesis or etiology. Feinstein (71) concluded that “An important principle in naming apparently new ailments is to avoid etiologic titles until the etiologic agent has been suitably demonstrated. A premature causal name can impair a patient's recovery from the syndrome, and impede research that might find the true cause.”

Presenting signs such as lower urinary tract signs (LUTS), which are often noted in patients with IC, have unfortunately affected the nosology of IC. The name given to any disease may reflect only a subset of the signs and problems associated with such a complex syndrome. By naming a disease for the organ associated with the primary presenting signs, we neglect the possibility that the disease may not originate from that organ, and many diseases can affect more than one organ. Different societies and organizations have created their own names and classifications for IC, leading to confusion and controversy among clinicians, patients, and researchers (72). Some of the names ascribed to IC are listed in Table 2 below. This review will use interstitial cystitis (IC) rather than one of the other names listed in Table 2 because of its heritage, familiarity, and common and Medical Subject Heading usage. Moreover, insurance systems in many countries or regions reimburse medical care for IC but not for its synonyms (80).

**Table 2:** Names applied to interstitial cystitis.

#	Name	Definition
1	Interstitial Cystitis (IC)	*High Sensitivity Definition*—pain, pressure, or discomfort in the pelvic area AND daytime urinary frequency 10+ or urgency due to pain, pressure, or discomfort, but not fear of wetting. *High Specificity Definition*—pain, pressure, or discomfort in the pelvic area AND Daytime urinary frequency 10+ or urgency due to pain, pressure, or discomfort, not fear of wetting AND symptoms did not resolve after treatment with antibiotics AND no treatment with hormone injection therapy for endometriosis. Exclusion criteria: bladder cancer, urethral diverticulum, spinal cord injury, stroke, Parkinson's disease, multiple sclerosis, spina bifida, cyclophosphamide treatment, radiation treatment to the pelvic area, tuberculosis affecting the bladder, uterine cancer, ovarian cancer, vaginal cancer, genital herpes, pregnancy (70, 73).
2	Chronic Primary Bladder Pain Syndrome (CPBPS)	Chronic primary visceral pain is chronic pain localized in the thoracic, abdominal or pelvic region, and is associated with significant emotional distress or functional disability. The distinct anatomical location is compatible with typical referral pain patterns from specific internal organs. The symptoms are not better explained by a diagnosis of chronic secondary visceral pain. Chronic primary visceral pain is multifactorial: biological, psychological, and social factors contribute to the pain syndrome. The diagnosis is appropriate, independent of identified biological or psychological contributors, unless another diagnosis would better account for the presenting symptoms (4) (ICD-11 MG-30.00).
3	Painful Bladder Syndrome (PBS)	Suprapubic pain, related to bladder filling accompanied by increased urinary frequency and nocturia, in the absence of proven urinary infection or other obvious pathology (74).
4	Bladder Pain Syndrome (BPS)	Chronic pelvic pain, pressure, or discomfort perceived to be related to the urinary bladder accompanied by at least one other urinary symptom: persistent urgency or urinary frequency (75).
5	Urologic Chronic Pelvic Pain Syndrome (UCPPS)	Idiopathic chronic pelvic pain of urologic origin in men or women. In Multidisciplinary Approach to the Study of Chronic Pelvic Pain (MAPP) Network studies, this term includes men and women with IC/BPS, or men with chronic prostatitis/chronic pelvic pain syndrome (76).
6	Interstitial Cystitis/Bladder Pain Syndrome (IC/BPS)	Chronic unpleasant sensation (pain, pressure, discomfort) perceived to be related to the urinary bladder, associated with lower urinary tract symptoms, in the absence of infection or other identifiable causes (76).
7	Hypersensitive Bladder (HSB)	Increased bladder sensation, usually associated with urinary frequency and nocturia, with or without bladder pain (77).
8	Central Sensitivity Syndrome (CSS)	A state of hyperexcitement of the central nervous system (CNS) involving the spinal and supraspinal structures due to an amplification of neural signaling involving various synaptic and neurotransmitter activities irrespective of a defined peripheral input (78, 79).

### Risk and protective factors for IC

Beginning when a patient encounters a clinician, precipitating, perpetuating, palliating, predisposing, and preventive factors may be identified. These risk and protective factors can be addressed clinically until a cause or reliably effective therapy is identified, and identifying predisposing and preventive factors can inform epidemiological studies and health promotion interventions (Table 3).

**Table 3:** Precipitating, perpetuating, palliating, predisposing, and protective factors related to IC across social, psychological, and biological domains [table structure adapted from (81–83)].

Domain	Precipitating	Perpetuating	Palliating	Predisposing	Preventive
Social/Environmental	• Recent adverse experiences • Interpersonal conflict • Low socioeconomic status^H^ • Unemployment^H^ • High healthcare utilization^H,C^ • Negative social support^H,R^ • Indoor confinement^C,R^ • Barren housing^C,R^	• Negative social support/esteem^H^ • Work/life conflict^H^ • Social stigma^H^ • Low socioeconomic status^H^ • Unemployment^H^ • Indoor confinement^C,R^ • Barren housing^C,R^	• Positive support from family, friends & healthcare givers • Supportive environments • Adequate financial resources • Effective environmental enrichment	• Early life adversity • Recent life adversity • Threatening environment • Low education level^H^	• Positive early life experiences • Positive recent life experiences • Safe environment • High educational attainment^H^
Psychological	• Poor coping style/problem solving • Maladaptive thoughts^H,C?,R?^ • Psychopathology/ • mental health problems (anxiety, depression, panic)^H, C?, R?^	• Negative coping style • Avoidance behaviors • Maladaptive thoughts^H,C?,R?^	• Resilient coping style • Prosocial behaviors • Predictability and perception of control	• Negative personality traits • Psychopathology • Low distress tolerance • Negative coping style	• Positive personality traits • Resilience • High distress tolerance • Positive coping style
Biological	• Illness/infection • Abuse or trauma • Physical symptoms/COPC • Natural disasters • Insufficient sleep/rest • Fatigue	• Increased central threat response system activity • Physical symptoms/COPC • Immune/inflammation dysfunction	• Normalized central threat response system activity • Physical activity • Sufficient sleep/rest	• Genetic susceptibility • Negative family health history • Old age • Discriminated race^H^ • Female gender^H^ • Susceptible breed^C, R^ • Female sex^C, R^	• Genetic resistance • Positive family health history • Adult • Race^H^ • Male gender^H^ • Resistant breed^C, R^

H , human; C, cat; R, rodent; No superscript, applies to all three species; COPC, chronic overlapping pain conditions.

#### Precipitating factors

Any unexpected sensory stimulus producing a meaningful experience of the world and of oneself can result in seeking medical care depending on the individual and the context in which the perception arises (84). Patient appraisals of sensory perceptions include their long-term implications based on the person's unique genetic and epigenetic history, memories of past events, environmental context, imagination of future possibilities (expectations), and perception of control (agency). Complex interactions between facilitating and inhibitory neuronal networks in the central nervous system (CNS) influence these perceptions (85, 86). These complex CNS interactions appear to be present in humans with chronic primary pain conditions like IC (85, 87) and have been reported in rodent models of IC (3).

#### Perpetuating factors

Many risk factors can also perpetuate IC (88, 89). The IASP explicitly acknowledges that “Pain is always a subjective experience that is influenced to varying degrees by biological, psychological, and social factors.” (6). Relevant social factors are species-dependent. We humans are an ultra-social species, the most social mammal on earth. Our health and well-being depend on the quality of our social connections, which share the importance of safety, food, and sex for our existence (90, 91). We have survived evolution by connecting with other humans to forage, hunt, and fight off enemies together (92). Social factors influencing patients with IC include the quality of their social connections, healthcare, and collective support (93).

Chronic pain can result in social isolation, loneliness, conflict, exclusion, and invalidation, and depression, anxiety, and post-traumatic stress disorder afflict some patients with IC that may result from the presence of such negative social factors (94). In 2023, a report by the United States Surgeon General described what he saw as an epidemic of loneliness in America and explained the health effects of loneliness and isolation (95). In contrast, cats have a facultative matrilineal sociality (96), and sociality varies widely across rodents, which may differ in group size, composition (e.g., juveniles, adult peers, mates), and the roles of specific, selective relationships (97).

Biological factors can also perpetuate IC. The recently proposed “extended autonomic system” consists of four complexly interrelated components: (1) the central autonomic network, (2) the autonomic nervous system with its three sub-systems: the sympathetic, parasympathetic, and enteric nervous systems, (3) neuroendocrine systems including the hypothalamic-pituitary-adrenal (HPA), sympathetic adrenergic, renin-angiotensin-aldosterone, and arginine vasopressin systems, and (4) immune/inflammatory systems (98). In the extended autonomic system, perceptions of threat accompany experiences that motivate escape or avoidance. They activate sympathetic, adrenocortical, and adrenomedullary activation, homeostatic resetting, and a perceived inability to cope that produces instinctively communicated signs. These responses also have been described as resulting from activating a central *threat* response system (CTRS) (99). Activation of the CTRS plays a clinically important role in IC symptoms in humans, cats, and rodents (See below) (100).

#### Palliating factors

Palliating, making a disease or its symptoms less severe or unpleasant without removing or knowing its causes, applies directly to IC because its cause remains unknown. And despite the identification of a host of pathophysiological variables *associated* with IC, none has yet led to generally effective *treatments* for IC in humans (60). The goal of protective palliating factors is to normalize the activity of the CTRS. Palliating factors include creating surroundings that provide safety, predictability, choice, and positive social support. In humans, psychosocial factors include positive support from family, friends, coworkers, and healthcare providers, supportive physical and work environments, adequate financial resources, and compensatory coping behaviors. Palliative biological factors include voluntary physical activity, mindful presence, good sleep hygiene, and a healthy, nutritious diet. In cats and rodents, environmental modifications that increase perceptions of safety, predictability, and control reduce perceptions of threat and can alleviate their clinical signs.

#### Predisposing factors

Predisposing risk factors are those that can increase susceptibility to IC. These include genetic vulnerability and family medical and mental health history (12), personality traits (101), early (ELA) (102, 103) and recent (RLA) life adversities (14), age, (104), race (105), and gender (106). In cats and rodents, predisposing factors include genetic vulnerability, ELA, personality traits, breed, sex, housing, and RLA (e.g., social conflict) (1, 107).

#### Preventive factors

Preventive factors are protective factors that can mitigate or counterbalance predisposing risk factors (81–83). These include genetic resistance, resilience-building early-life experiences, positive psychological factors, and strong, positive social connections. Although generally less modifiable than precipitating, perpetuating, and palliative factors, identifying predisposing and preventive factors may facilitate early identification of susceptible individuals, regardless of species, to permit earlier preventive and therapeutic interventions (108) in addition to informing epidemiological studies and health promotion interventions.

## Discussion

### IC in human beings

#### Background

Although IC has been assumed to be a bladder disorder for centuries (109, 110), recent literature reviews of cystoscopy studies reported finding no convincing evidence that glomerulations (punctate petechial hemorrhages observed after hydrodistension) should be included in the diagnosis or phenotyping of IC since they did not correlate with symptoms and were found in patients without IC (111). Based on these results, the 2023 report of a committee of the International Continence Society concluded that “clinically at this time, the presence or absence of glomerulations on cystoscopy should not be used as a diagnostic or prognostic criterion for IC….” (112). Another recent study also cast doubt on the usefulness of bladder biopsy for the evaluation of severity in women with non-Hunner lesion IC (113). Moreover, a 2022 systemic review of uncontrolled studies of surgical interventions for IC found that fulgurating lesions in humans resulted in only a 31%–54% reduction in painful bladder symptoms, and no significant decrease in symptoms was noted in patients after a partial cystectomy with augmentation ileocystoplasty, suggesting the etiology of IC is more complex than one isolated to the bladder for many patients (114).

#### Precipitating factors

A variety of risk factors that can precipitate IC have been reported in women, including early symptom onset, symptoms increasing the week before menses, symptom flares after sex, and a family history of similar bladder symptoms (104). Additionally, ELA and RLA have been investigated as potential risk factors for IC. For example, Warren et al. (115), identified both prodromal (antecedent) and non-prodromal presentations of IC, and Lutgendorf et al., have reported inflammation and HPA abnormalities in women with IC (14). In their study, 154 women with IC and 32 healthy controls completed surveys, collected salivary cortisol, and provided a blood sample for analysis of seven lipopolysaccharide (LPS)-stimulated cytokines and chemokines. Patients with greater exposure to RLA or cumulative ELA and RLA of at least moderate severity had increased concentrations of a composite of all cytokines, but nocturnal cortisol and cortisol slope were not associated with RLA or inflammation. This altered stress response characterized by increased cytokines and lack of a cortisol response could contribute to the signs often noted in IC patients—urinary urgency, frequency, bladder or voiding pain, and painful intercourse, supporting the importance of adverse life events as risk factors for IC via a biological mechanism.

#### Perpetuating factors

As mentioned, the severity of IC is a perpetuating risk factor. Interstitial cystitis can vary in severity from mild to severe across three dimensions: social interference, psychological distress, and strength of intensity. Hay, et al. (116), recently proposed numerical rating scales for social interference—“How much did the pain interfere with your activities in the last week on average?”, psychological distress—“How much distress did you experience in the last week because of your pain on average?”, and biological strength of pain intensity—“How strong was your chronic pain in the last week on average?” on an 10-point scale ranging from 0 (no pain-related interference, distress, or strength) to 10 (unable to carry on activities, extreme pain-related distress, or worst pain imaginable). Considering all these dimensions can help differentiate between low and high-impact pain in IC patients, which may influence approaches to care and outcomes.

An additional perpetuating factor in humans is increased bladder permeability (28). Although commonly associated with IC, the cause of the increased permeability is unknown. Increased permeability led to attempts to use glycosaminoglycan compounds like pentosan polysulfate (Elmiron®, PPS) as a treatment for IC (117). Recent research showing cross-communication among the bladder, bowel and other organs, nerves, cytokine-responding cells, and the nervous system has led to the suggestion that IC may reflect the presence of a more complex systemic disorder (28).

#### Palliating factors

Although much IC research focuses on subjects with relatively severe symptoms, studies of IC patient outcomes in the years following diagnosis report that most patients have clinically relevant (≥50%) symptom improvement (63–65). Palliating factors can help patients live well with chronic pain, including IC. For example, a grounded theory study of 17 middle-aged women and men reported chronic pain severities similar to those found in primary care patients but with interference and emotional burden scores below the US national average (118). The subjects reported that their main problem was integrating their present experience with past experiences and motivations and finding meaning in their present experience to regain a sense of well-being and self-fulfillment to experience life meaningfully, even in difficult situations. Resolving this problem required them to make personal sense of their pain, to re-occupy their selves, and then to decide to turn their life from patient to person. This turn could be *facilitated* or *hindered* by interactions with clinicians and social others (family, friends, co-workers) and one's occupational drives, the things people need to, want to, and are expected to do in life. These steps were followed by a lifelong maintenance process of engaging in the everyday activities that other people do in families and communities to occupy time and bring meaning and purpose to their lives, which allowed them to live well despite their pain (118).

#### Predisposing factors

Predisposing risk factors that can increase susceptibility to IC include genetic vulnerability (12), family (104, 119) medical (120) and mental health history (121), personality traits (122), ELA and RLA (14), age (119). and gender (123). Associations between ELA and IC symptoms have been reported (13, 103, 124). A recent case-control study comparing women with IC (cases) with healthy controls found that cases had higher median numbers of ELA, with greater occurrence of abuse (emotional/physical/sexual) and household challenges. Cases had a seven-fold increased odds of having four or more ELA compared with controls (13).

#### Preventive factors

Preventive factors that can mitigate or counterbalance previously described risk factors include genetic resistance, resilience-building life experiences, positive psychological factors, and strong, positive social connections. Early identification of risk factors increases the time available for implementing effective protective measures (108). Assessing protective and ELA experiences is becoming more common in clinical practice in some regions; time will tell how effective such efforts are in identifying and implementing therapeutic strategies (125).

### IC in cats

#### Background

Based on the many similarities between IC in humans and cats, replacing FIC with IC in cats as an initialism for either feline interstitial or idiopathic cystitis seems to be a reasonable nosological description of cats presented with cLUTS in the presence of compatible urinalysis and imaging findings that avoids an etiologic title. Cystoscopic or other advanced imaging evaluations could be considered for further investigation of cats that do not respond to treatment since many other causes of cLUTS have been reported in cats (126). However, similar to humans, cystoscopic findings (e.g., glomerulations) are not pathognomic for FIC, and lesion severity does not correlate with the severity of clinical signs (61).

#### Precipitating factors

Approximately 1.5% of cats presenting at veterinary practices exhibit one or more LUTS (127, 128). Poor outcomes for cats with elimination behaviors deemed inappropriate by owners are common and include euthanasia or relinquishment to shelters (129, 130). In cats with urethral obstruction due to IC, recurrence rates of 17.0%–58.0% have been reported (131). Rates of euthanasia have been reported to range from 8.5% and 26% in cats diagnosed with IC (132, 133).

Variable combinations of problems with the gastrointestinal, endocrine, and cardiovascular systems and fearful, anxious, or aggressive behaviors are commonly reported in cats with IC (134–136). Immune activation and pro-inflammatory cytokine release that can lead to SB have been reported in cats with IC (137, 138). Sickness behaviors are a well-documented group of non-specific clinical signs thought to reflect a change in motivation to conserve energy for recovery from infection (139) that have been reported in cats (16) and other species, including rodents (140). In cats, SB include anorexia or decreased appetite, vomiting of food, hair, or bile, eliminating outside of the litter pan, decreased social interactions and grooming behavior, and increased frequency and intensity of attempts to hide (16, 25, 141, 142).

Like humans with IC, recent adverse events are also a risk factor for cats with IC. Sickness behaviors in cats have been reported in response to environmental stressors, including changes in husbandry routine and caretakers and discontinuation of EE. These events resulted in a 3.2-fold increased relative risk for SB during weeks when unusual external events occurred compared to control weeks (16). Many cats also display SB before their first or recurrent episodes of LUTS, so identifying and monitoring SB may allow caretakers to intervene early to prevent the development of cLUTS.

#### Perpetuating factors

As with humans, the severity of clinical signs of IC in cats can vary from mild to severe and result in recurrent episodes of LUTS. Increased bladder permeability is also a perpetuating factor in cats (29), and the association also led to unsuccessful attempts to use PPS to treat IC in cats (143–145). Psychological distress can be affected by the quality of the housing environment, especially for cats strictly confined indoors. Cats likely experience the home environment very differently from humans' experience of it, so they may perceive many environmental features as aversive or stressful. Environmental factors that can influence cats' perception of threat include the quality of human-cat relationships and interactions with conspecifics and other animals (e.g., dogs) in the cat's social environment and their perception of predictability and control of their surroundings (142, 146–149). Cats with IC may respond with fearful, anxious, and/or aggressive behaviors toward their owners that can negatively impact the human-animal bond, feeding into a negative feedback loop that may exacerbate cLUTS and result in poor outcomes for the cat, including euthanasia.

#### Palliating factors

The number, frequency, and severity of episodes of IC may depend on several factors, arguably the most important being the dedication of the owner to reducing the cat's perception of threat by providing varied opportunities to engage in highly motivated, species-specific activities, cognitive challenges, and sensory experiences (62, 150, 151). Effective Multimodal Environmental MOdification (MEMO), designed to provide safety, predictability, and perception of control to confined cats, has been reported to reduce cLUTS and related signs of IC in both research and clinical studies of cats with IC (16, 62).

#### Predisposing factors

Many risk factors predispose cats to IC. Cat factors include male sex (107, 152–154), neutered (152, 155), overweight (107, 128, 155, 156), purebred (10, 107), middle-aged (11, 153), and fearful or anxious (128, 154). Environmental risk factors include indoor confinement (154, 156–158), low litter-box-to-cat ratio (152, 156, 157), and inadequate access to elevated resting areas or vantage points (128, 155). Other risk factors include decreased activity and opportunities to engage in species-typical predatory behaviors (156, 157), and social conflict within the home (107, 128, 155, 156). Indoor-housed cats have little or no control over who their social partners are, how much space they can put between themselves and others, the type, amount, or availability of food, or the quality or quantity of environmental stimuli, including lights, noise, odors, and temperature (159).

#### Preventive factors

The current recommendation for preventing recurrent signs of IC is to decrease the cat's perception of threat using MEMO (62). The provision of an enriched environment containing abundant resources optimally placed is the “gold standard” for all indoor-housed cats to ensure adequate welfare (160). This includes providing choice wherever possible, especially when introducing or changing essential resources such as resting areas, diet, litter, or litter boxes.

### Rodent models of IC

#### Precipitating and perpetuating factors

Bladder-centric, physical, and psychological stressors are all used to study precipitating and/or perpetuating risk factors. Early bladder-centric rodent models have been reviewed (161). More recent models instill different noxious compounds, such as protamine sulfate, cyclophosphamide (intraperitoneally), acids, zymosan, and LPS, which can increase urothelial permeability, induce an inflammatory response, alter visceromotor responses, and inflict acute pain depending on the compound and dose. Additionally, treating neonatal rats with zymosan increased bladder plasma extravasation and urinary frequency in them as adults, demonstrating that neonatal bladder injury, an adverse early life experience, can increase urothelial permeability and bladder sensitivity in adults subjected to injury early in life (3).

Psychological and physical stressor models more accurately mimic naturally occurring IC. For example, an early psychological model applied to female mice found that moderate stress induced by prolonged illumination resulted in the detachment of urothelial tight junctions and the loss of the bladder permeability barrier, leading to expanded intercellular spaces among urothelial cells. Removal of the stressor led to rapid restoration of new tight junctions, preventing long-term malfunction of the blood-urine barrier (162). In an early model of physical stress (163), immobilizing adult male Sprague/Dawley rats for 30 min in a plexiglass immobilizing tube activated over 70% of bladder mast cells. Moreover, mast cell progenitors can be programmed toward a hyperactive phenotype and increased tissue activation by ELA, which may explain their presence in mast cell-related disorders like IC in adulthood (164, 165).

The psychological stressor water avoidance stress (WAS) model was developed to investigate the role of chronic stress as a perpetuating risk factor for visceral pain symptoms in patients with functional gastrointestinal disorders (166). In this model, a single rodent is placed on a small platform centered in the middle of a water-filled basin, usually for one hour each day for ten days. Rodents naturally avoid entering the water, which increases their perception of threat when no escape is possible. Exposure to WAS increases anxiety-like behaviors, urinary frequency, and bladder hyperalgesia. Histological changes in bladder tissue from WAS-exposed rats included ulcerated areas, edema, vascular congestion, inflammatory cell infiltration, increased angiogenesis and mucosal mast cell numbers (2), and increased bladder mucosal permeability in female Wistar rats (167). Additionally, Wang et al. (168), reported greater activation in brain regions of the central micturition circuit and increased engagement of portions of the micturition circuit responsive to urgency, viscerosensory perception, and its relay to motor regions coordinating imminent bladder contraction in adult female Wistar-Kyoto rats subjected to WAS to model IC.

Another psychological stressor is social defeat, which has been used to mimic the physiological and behavioral abnormalities resulting from the social conflict between individuals (169). For example, West, et al. (170), exposed adult male C57BL/6JArc mice to aggressive male ex-breeder Arc mice for one hour each day for 10 days. The social defeat mouse was placed into physical contact with the aggressor for a maximum of five minutes and then separated by a transparent perforated barrier for 55 min. This resulted in significant increases in plasma corticosterone concentrations and significant decreases in voiding frequency in the socially defeated mouse. Nerve-evoked bladder contractile responses were significantly increased in bladders from socially defeated mice at all frequencies, resembling changes after partial bladder outlet obstruction (2). This social defeat model has proven difficult to implement in female mice because, under most conditions, neither male nor female resident mice will attack intruder females (171), so alternatives have been proposed (172, 173).

#### Palliating factors

Studies in rodent models have also tested the effects of EE after threat induction. Like MEMO therapy for cats, EE aims to improve health by providing more complex and novel living conditions than standard housing offers by adding inherently enriching physical and/or social elements into animals' environments (174–177). Animal models of EE have been used for more than seventy years to promote healthy physiological responses (176). Physical EE includes providing home cages that are larger and more complex and objects that promote activity, play, nesting, and/or foraging to promote cognitive stimulation and physical activity, whereas social enrichment is implemented by housing animals together to provide opportunities for them to engage in species' typical social behaviors like social play, communal nesting, cooperative feeding, allogrooming, and nursing (175). Studies incorporating EE that more closely reflect the umwelt (178) of the animals studied seem to offer more ecologically valid models of IC in humans and cats (99).

Voluntary wheel running reportedly improves both physical and behavioral abnormalities in induced models of IC (179–182), as does EE in models of visceral pain after ELA (183–186). For example, maternal separation for six hours each day from postnatal day 1–21 resulted in visceral pain, anxiety, and depression in adult male C57BL/6J mice, which was effectively prevented by EE interventions in prepuberty and puberty (183). Environmental enrichment was more effective in male mice, whereas longer exposure to EE was required in female mice to demonstrate positive effects (184). In another study of EE, normal male and female Wistar rat pups received subcutaneous administration of LPS (50 µg/kg) 3 and 5 days after birth to induce an inflammatory response, after which they received EE from post-natal day 25–120. The EE increased motor and search activity and contextual conditioned fear in both sexes and reduced anxiety in males and depressive-like behavior in females. In contrast, LPS prevented the beneficial effects of EE on anxiety in females and on conditioned fear in both sexes (185). The systemic effects of EE on conditioned fear reactions have been reviewed (187).

In a 2024 study, adult Fischer-344 rats of both sexes were exposed to WAS daily for seven days. After confirming the rats had developed visceral hypersensitivity, they were housed in environmentally enriched cages with ample bedding, toys, food enrichment, and burrowing tunnels in groups of four rats/cage for two weeks, after which visceral and somatic sensitivity was assessed, followed by collection of colon tissue to measure permeability. In both sexes, EE reversed stress-induced visceral and somatic hypersensitivity and colonic hyperpermeability to control animal levels, suggesting that EE can ameliorate stress-induced pathophysiology (186).

#### Predisposing factors

Models of predisposing risk factors like ELA are relevant because ELA is common among humans and cats, and childhood maltreatment is a particularly potent risk factor for IC (102). In the United States, at least 1 in 7 children experienced child abuse or neglect in 2020^2^, and about ***1 in 20 boys and 1 in 4 girls*** experience sexual abuse during childhood^3^. This terrible prevalence is similar to the reported worldwide pooled overall rate of 21%–27% (188). Moreover, the prevalence of sexual abuse ranged between 1.3% and 50% in patients presenting with LUTS in a recent (2023) systematic review, which reported that urinary storage symptoms, voiding difficulties, voluntary holding of urine, and urinary tract infections were associated with psychosocial threats, depression, and anxiety (189). Among cats, orphaning and sheltering are common early adverse life events (190).

Animal models of ELA include limited bedding and maternal separation (191–193). In a study of limited bedding, female and male neonatal Wistar rats were exposed to limited bedding on postnatal days 2–9. Then, at 10–11 weeks, they underwent colorectal distension and abdominal electromyography, and cerebral blood flow was measured. Exposure to limited bedding increased abdominal electromyography responses to colorectal distension in rats of both sexes, whereas functional brain responses differed across circuits involved in the regulation of pain and emotion; responses were greater in female rats in the locus coeruleus/lateral parabrachial nucleus complex and brain noradrenergic system (the CTRS). In models of maternal separation, female (179) and male (182) C57/Bl6 mice were separated into clean glass containers with small amounts of their home cage bedding material and held for three hours on postnatal days 1–21. Maternal separation increased bladder sensitivity, void frequency, and mast cell degranulation and reduced corticotropin-releasing factor receptor 1 and glucocorticoid receptor mRNA levels when assessed four weeks later.

Early life adversity can trigger evolutionarily conserved survival responses that include epigenetic modification of gene expression in patients with IC and other primary chronic pain conditions (102, 194). Preclinical and clinical epigenetic findings include changes in DNA methylation, histone modification, noncoding RNA, RNA modification, and chromatin remodeling factors. The contribution of these mechanisms to trait variability after ELA and how such mechanisms might lead to identifying new therapeutic options has also been reviewed (195).

Recent life adversity and ongoing chronic threats have also been associated with neuroendocrine and inflammatory alterations in patients with chronic pain, including IC and related conditions (14). Animal models of RLA include WAS, social defeat, and various other threat-inducing events. Each has been tested in rodents of different ages, sexes, and strains, resulting in various LUTS, and each has advantages and disadvantages for understanding IC (2, 3).

Early and recent life adversity have also been combined in what are called “two-hit” models of IC, since patients with IC commonly have histories of both predisposing (ELA) and precipitating (RLA) risk factors (196). By itself, ELA is neither necessary nor sufficient to lead to IC but may potentiate susceptibility to adverse outcomes when a susceptible individual is exposed to threats later in life. These models also have limitations, as recently discussed (196).

### Implications of the “5 Ps” risk and protective factor approach to IC for clinical care

#### Precipitating and Perpetuating factors

IC patients usually present with variable combinations of signs and symptoms resulting from underlying risk factors described previously, which are identified from the signalment and a careful history. Skepticism from medical providers can diminish patient satisfaction and outlook (197, 198), and a qualitative study reported that women with IC commonly experience disbelief and pain dismissal from healthcare providers, which can prevent effective communication with them and lead to suboptimal care (199).

#### Palliating factors

Providing trauma-informed care (200), identifying and mitigating identifiable risk factors to the extent possible, and providing positive, meaningful social connections and support (protective factors) can be therapeutic (201, 202). The authors of the qualitative study mentioned above proposed adopting a patient-centered approach by inviting patients to share their personal experiences, beliefs, and embodied knowledge during treatment discussions to elevate the interaction beyond typical urology considerations by affording patients the ability to share in decision-making when no clear “best” treatment option exists. They argued that appreciating the struggles, anxieties, and gendered inequities that women with IC experience during their diagnostic journey could enrich patient-centered treatment and improve the well-known delay in making the diagnosis of IC (199).

Although trauma-informed care is not specifically mentioned in all pelvic pain guidelines, clinicians are encouraged to listen attentively, convey interest and compassion, and validate patients’ symptom experiences (200). Screening for a family history of COPC beyond first-degree relatives may be useful during the initial evaluation of patients presenting with pain, anxiety, or depression because it could lead to earlier diagnosis of other relatives who could benefit from treatment (12).

Positive relational social support systems can also improve patient health (203), and IC patients may not receive enough support from their usual social support systems, given the general lack of understanding about their condition (204–206). The U. S. Surgeon General has identified various ways to improve social connections and health. When available, multidisciplinary pain clinics and positive support groups can provide social connections to help scaffold patients' recovery of positive social attachments.

Non-pharmacological psychological therapies have been recommended for IC, although their effectiveness is currently limited (60). Moreover, the most recent (2020) Cochrane review of psychological therapies for chronic pain in adults reported that, on average, people treated with cognitive behavior therapy probably experience slightly less pain and distress by the end of the treatment and six to 12 months later (moderate-quality evidence), may experience slightly less disability on average (low-quality evidence), and probably experience very slightly less pain and distress (moderate-quality evidence), but levels of disability may be similar to those of people who received a non-psychological treatment (low-quality evidence) (207).

As mentioned, biological treatments for IC have also met with little success (60). Schrepf, et al., recently reported that IC pain might have a treatable neuropathic component (208), but a recent, adequately powered randomized controlled trial of women with chronic pelvic pain without identified underlying disease reported gabapentin (used to treat some neuropathic pain conditions) to be equivalent to placebo in patients randomly assigned to receive gabapentin (titrated to a maximum dose of 2,700 mg daily) or matching placebo for 16 weeks (209). Gabapentin also resulted in significantly more self-reported serious adverse events (7%) than the placebo (2%).

Parsons' recommended approach to women presenting with symptoms of IC could be updated and combined with the compassionate assurance of ongoing vigilance and care to help prepare patients to begin evidence-based palliative approaches to care (104). In cats with IC, such a tailored case-specific approach (MEMO) has been successfully used to reduce clinical signs and related comorbidities for over three decades (16, 62) and is the current standard of care in veterinary medicine (210, 211). Cats with IC respond to effective environmental modification *without any treatments directed toward the bladder*, as do rodents subjected to physical and psychological stressors, emphasizing the role of the CTRS in IC. In contrast, people with IC are regularly given bladder treatments regardless of their demonstrated equivalence with a placebo (60). The placebo (regression to the mean and other statistical factors, and positive expectation) response rate among IC patients is reportedly 50% or more, suggesting that the CTRS plays a clinically important role in IC symptoms in humans as it does in cats and rodents (212). In fact, Parsons et al. (23), administered oral PPS, a treatment later demonstrated to be equivalent to a placebo^4^ (117), to 24 patients with IC. Within 4–8 weeks of initiation of therapy, 20 of the 24 patients experienced a decrease of at least 80 percent in pain, nocturia, and urinary urgency (excellent response), and 2 experienced a 50–80 percent reduction of symptoms (good response). The excellent overall success rate of 92 percent persisted during up to 24 months of follow-up despite the patient's history of longstanding, severely symptomatic disease. It is no surprise that Parsons et al., believed PPS to be an effective oral treatment for IC.

Examples of tools to diagnose chronic pain type, assess physical functioning, emotional functioning, patients' beliefs and coping, multidimensional measures, and pharmacological treatments based on pain classification have recently become available in an open-access practical guide to Recognize, Assess, Treat, and Evaluate (RATE) patients with chronic pain seen in primary care (213). The guide also describes how to use this information to tailor treatment plans to meet individual patient's needs and to evaluate patients and the impact of treatment plans over time, with a particular focus on strategies to improve the patient's ability to self-manage their pain and related symptoms and to perform daily functions despite persistent pain. The article explicitly addresses chronic primary pain and could be adapted to IC and related conditions by acknowledging previously discussed pharmacological limitations.

Additional resources are also available; Darnall et al. reviewed several promising education and treatment technology innovations to improve access and scalability of evidence-based behavioral pain treatments (214). Identifying risk and protective factors early, in primary care if possible, might also shift the balance of perpetuating factors to palliating and protective factors to promote recovery.

### Implications for research

#### Social and psychological palliating and protective factors

Research is just beginning to study the value of positive social connections for people with chronic pain (215). Despite the centrality of the effects of human sociality on our survival as a species, research into the effects of social factors on chronic pain in general and IC in particular remains in its infancy. Notwithstanding the remarkable work of some, much more remains to do. Social research that may be relevant to IC is starting to be published. For example, Groups 4 Health (G4H) is an emerging therapy program that targets social group disconnection (216). A recent study comparing G4H with treatment as usual for people with chronic low back pain (another COPC) found G4H to be significantly more effective in reducing pain intensity and disability post-intervention and at a one-month follow-up. More robust treatment group identification and cohesion early in the program among G4H recipients predicted better pain outcomes over time, which was fully mediated by perceptions of personal control (217).

The acceptability of G4H to clients and therapists and its feasibility for wider implementation have also been evaluated (218). Both client and therapist satisfaction was high, with all average ratings more than 70% positive, significantly exceeding the cognitive behavior therapy client comparison group. Retention was greater than 80%, and fewer than 10% of clients said that they had not attempted the homework. Therapists and clients both emphasized the contribution of the group context itself as a vehicle to achieve positive outcomes.

Other recently developed psychological therapies, such as emotional awareness and expression therapy (219), pain reprocessing therapy (220, 221), and cognitive functional therapy (222), have also shown promise for COPC, including IC (223). Although the studies need to be replicated in larger populations with longer follow-ups, effective therapies should also reduce the severity of associated comorbidities, as has been reported in humans with IBS (224) and cats with IC (62).

#### Biological factors

Evaluating the conserved transcriptional response to adversity (CTRA) or Toll-like Receptor-4-stimulated cytokines in the blood of patients with IC and other COPC might provide useful markers of CTRS activity and response to therapeutic interventions in both human and animal studies. The CTRA is a pattern of gene expression that reflects the activation of pro-inflammatory pathways and the suppression of antiviral and antibody-related pathways in response to stress, trauma, or social isolation (225). The CTRA has been linked to various adverse health outcomes, such as increased risk of infection, inflammation, and chronic disease. It also appears to be responsive to effective therapy, which may permit its use in clinical studies of palliative and other complementary approaches to care (91). Toll-like Receptor-4 (TLR-4) receptors can trigger an inflammatory response to bacterial components like LPS and immune stimulation by the CTRS in patients with IC and related COPC. Research using the blood of patients with IC and healthy controls has found that patients with IC have higher levels of TLR-4-stimulated cytokines and chemokines. TLR-4 may also interact with other factors, such as ELA or RLA, to increase the risk or severity of IC (14, 226).

Increased heart rate and changes in heart rate variability are also related to chronic primary pain conditions like IC (54). Dudarev et al. (227), recently reported a positive predictive relationship between increased sleep heart rate and next-day pain intensity in patients with two chronic primary pain conditions, fibromyalgia (228) and primary back pain (229), with no interaction between the types of chronic pain. These findings support the hypothesis that threat-driven increases in CTRS-mediated autonomic hyperactivation precede increases in next-day pain intensity in patients with chronic primary pain conditions.

## Conclusions

The IASP's recent classification of IC and related conditions and the expansion of the dimensions of the effects of chronic pain on people's lives has thus afforded a unique opportunity to rethink these problems from a cause to a risk and protective factor perspective. The interconnected body-brain-mind creates the “organismic experience” of chronic pain. The standard urological explanation is that the experience of IC is initiated “bottom-up”, either by some abnormality of urothelial permeability that results from and/or leads to the absorption of toxins from the urine that results in increased nociceptive input to the central nervous system, or, more recently, by low bladder capacity (230). This input results in systemic and local bladder abnormalities and can result in pain, which activates the CTRS and its output to the peripheral adrenocortical, autonomic, and immune systems, creating a feedback loop that perpetuates the pain and results in IC's waxing and waning course.

More recent research has shown that the experience of IC can be initiated from the “top-down” by the persistent perception of environmental threat and/or a sensitized CTRS, leading to the activation of peripheral adrenocortical, autonomic, and immune systems, resulting in systemic and local bladder abnormalities and creating a feedback loop that perpetuates the pain and results in IC's waxing and waning course. To date, evidence from studies of both human and feline patients with IC, and rodent models of physical and psychological stressors lend more support to the “top-down” perspective. Moving the source of IC pain to the brain as a motivational state resulting from central sensitization rather than peripheral nociceptive input offers both clinicians and researchers novel opportunities to improve care for and study of IC for the better. It may even be that IC results from an excess of risk to protective factors, making this imbalance a cause rather than a consequence of IC.

## Footnotes

^1^https://www.iasp-pain.org/advocacy/definitions-of-chronic-pain-syndromes accessed March 18, 2024.

^2^https://www.cdc.gov/violenceprevention/childabuseandneglect/fastfact.html Accessed March 18, 2024.

^3^https://www.cdc.gov/violenceprevention/childsexualabuse/fastfact.html Accessed March 18, 2024.

^4^https://beta.clinicaltrials.gov/study/NCT00086684?cond=Interstitial%20Cystitis&intr=elmiron&rank=2 Accessed March 18, 2024
